# Effects of microbial interspecies relationships and physicochemical parameters on volatile flavors in sorghum‐based fermented grains during the fermentation of Shanxi light‐flavored liquor

**DOI:** 10.1002/fsn3.3185

**Published:** 2022-12-15

**Authors:** Aiguo Luo, Niutian Yang, Jing Yang, Jianwei Hao, Jia Zhao, Shengli Shi, Bianfang Hu

**Affiliations:** ^1^ Department of Biological Science and Technology Jinzhong University Jinzhong China; ^2^ Shanxi Higher School Solid Fermentation Engineering Technology Research Center Jinzhong University Jinzhong China; ^3^ Food Science and Engineering Shanxi Agricultural University Jinzhong China; ^4^ School of Life Science Shanxi University Taiyuan China

**Keywords:** light‐flavored Baijiu, microbial community, physiochemical, sorghum‐based fermented grains, volatile substances

## Abstract

In this study, high‐throughput technology was used to reveal the core microbial community in sorghum‐based fermented grains during different fermentation periods and to quantify the impacts of physicochemical parameters and microbial interspecies relationships on the volatile flavors. Headspace solid‐phase microextraction, coupled with gas chromatography–mass spectrometry, was used to select 14 major volatile products with relative content greater than 1% in at least one sample, including three alcohols, one acid, eight esters, and two alkanes. The relative content of alkanes was only high on the first day and continued to decrease during the later fermentation stage. As fermentation progressed, the relative content of ethanol, ethyl acetate (aroma), and isoamyl alcohol (pungent, spicy) first increased and then decreased. In addition, the relative content of other ethyl esters continued to increase. In the early stage of fermentation (1–7 days), the temperature, moisture, and alcohol content showed an upward trend, while the content of reducing sugar decreased. As the temperature decreased in the middle and later stages (7–28 days), the physicochemical parameters tended to stabilize. In community composition, the dominant bacterial genera were *Lactobacillus*, *Streptomyces*, and *Acetobacter*, and the fungal genera were mainly *Issatchenkia*, *Torulaspora*, and *Pichia*. Network analysis identified a total of 10 core microbiota as the main contributors of esters and alkane metabolites. Moreover, total acidity and reducing sugar played important roles in promoting the formation of core microbiota and succession of dominant taxa.

## Graphical abstract

In this study, high‐throughput technology was used to reveal the core microbial community in sorghum‐based fermented grains during the different fermentation periods and quantify the impact of physicochemical parameters and microbial interspecies relationships on the volatile flavors.

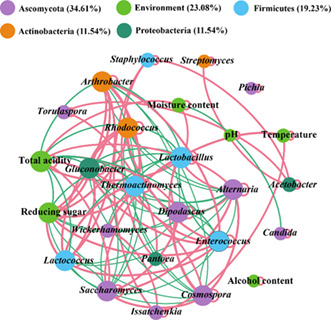

## 1 INTRODUCTION

Baijiu is a traditional Chinese distilled liquor made from natural mixed cultures and solid‐state fermentation. According to the flavor characteristics, it can be divided into three types: sauce flavored, strong flavored, and light flavored (Pang et al., 2018). Light‐flavored Baijiu is the oldest and most widely consumed type. It is produced from the fermentation of sorghum, with Daqu as the fermenting agent, via traditional fermentation technology and solid‐state fermentation (Huang et al., 2020). Fermented grains represent a complex and unique ecosystem, and they are the primary microbial habitats, biochemical reaction systems, and direct sources of flavor compounds for Chinese liquor production. Two fermentation processes of fermented grains include: the initial fermentation, using sorghum as the raw material and Daqu and water for solid‐state fermentation, called the Dacha fermentation; and the second fermentation, using the substrates of the first fermentation and distillation as the raw material to add Daqu for solid‐state fermentation, called the Ercha fermentation. During production, there are obvious differences between the Dacha liquor and Ercha liquor. For example, the Dacha liquor is mellow in flavor and aroma, while the Ercha liquor is rich in acid and ester aromas. These changes are directly linked to the fermentation substrate, microbial flora structure, flavor compound development, and fermentation environment (Wang et al., 2021).

During the fermentation of light‐flavored Baijiu, microorganisms adapt to the environment to promote their growth and metabolism. The physicochemical indicators in the fermentation environment can affect the metabolic growth of microorganisms in the fermented grains, and the metabolic growth of microorganisms also affects the microbial community structure and, thus, the flavor profile. Therefore, studying the changes in microbial flora structure and physicochemical parameters during the fermentation light‐flavored Baijiu is of great significance for understanding the Baijiu brewing process. In recent years, there have been numerous studies on light‐flavored liquors that mainly focused on the structure of the microbial flora and functional strains, and the influence of flavor substances on the style of liquor. For example, Wang et al. (2015) studied the bacterial and fungal communities in Daqu liquor. Wang et al. (2019) studied the microbial community structure during the brewing process of Sichuan Xiaoqu liquor. Xue et al. (2021) analyzed the bacterial diversity, organic acids, and flavor differences during the fermentation of Dacha liquor and Ercha liquor. However, the dynamics of the physicochemical properties, microbial community, and flavor compounds of fermented grains are still not fully understood. In particular, the effect of different fermentation periods is rarely considered. In recent years, a high‐throughput sequencing method, which does not rely on pure culture, has been used to analyze microbial community diversity in environmental samples. The technology does not require the construction of clone libraries, eliminating the need to screen the libraries. This not only simplifies the experimental steps but also greatly improves the sequencing efficiency (Zhang, Zhao, et al., 2014), providing an opportunity to study the microbial composition in fermented grains.

The fermented grain samples used in this study were from Shanxi Province, including light‐flavored liquor fermented with local sorghum as the main ingredient. Subsequently, the microbial community structure, reducing sugar content, alcohol content, and other physicochemical parameters of fermented grains were determined during different stages of fermentation. The dynamic changes in microorganisms, metabolites, and physicochemical indicators were analyzed, and the cooccurrence network was used to characterize the contribution of microorganisms to metabolites and the impact of physicochemical parameters and microbial interspecies relationships on the volatile flavor profiles. The results of this study provide a theoretical basis for clarifying the brewing mechanism of Baijiu to improve the quality and fermentation efficiency of light‐flavored liquors.

## 2 MATERIALS AND METHODS

### 2.1 Sample collection

Fermented grains were collected from the Shanxi Baozi distillery. The multipoint sampling method was used to take equal amounts of fermented grain samples from the upper, middle, and lower layers of the fermentation tank. The samples were mixed evenly and sealed in sterile bags. One bag was stored in a −20°C for determination of physicochemical indicators, and the other was stored at −80°C for DNA extraction. The samples of fermented grains fermented for 1, 7, 15, 21, and 28 days (abbreviated as DJP1d, DJP7d, DJP15d, DJP21d, and DJP28d, respectively) by Daqu were used for physical and chemical analyses, volatile flavor analysis, and sequencing analysis. Each sample was measured in triplicate, and each experiment was repeated three times. A total of 90 samples were used for analysis.

### 2.2 Determination of physicochemical parameters and volatile flavor substances

The temperature of the fermented grains was measured by inserting a thermometer (RE‐W5007; People's Electric Group Co.) into the pit at a depth of 1 m. The pH of the fermented grains was determined using a pH meter (Model UB‐7; Denver Instrument Company), and the total acidity was determined using acid–base titration according to the national standard for food safety. Alcohol content was measured using the alcohol meter method (Alcolyzer Wine; Anton Paar Co.). The reducing sugar was determined via direct titration according to a previously published method (Kang et al., 2022). The moisture content was determined using the constant‐temperature drying method. The concentration of volatile flavor substances in fermented grains was measured via headspace solid‐phase microextraction coupled with gas chromatography–mass spectrometry (Clarus 600; PerkinElemer; Pang et al., 2018).

### 2.3 DNA extraction, PCR amplification, and Miseq sequencing

DNA was extracted from each sample according to the instructions of the E.Z.N.A.® soil DNA Kit (Omega Bio‐Tek). For bacteria, the V3–V4 region of 16 S rDNA was amplified with primers 341F (5’‐CCTACGGNGGCWGCAG‐3′) and 806R (5’‐GACTACHVGGTATCTAATC‐3′). For fungi, the ITS1–ITS2 region was amplified with primers ITS1F (5’‐CTTGGTCATTTAGGAAGTAA‐3′) and ITS2R (5’‐GCTGCGTTCTTCATCGATGC‐3′). The PCR reaction system consisted of 15 μl 2 × Hieff® Robust PCR Master Mix, 1 μl each of forward and reverse primers, and 10 ng of template DNA. The total volume of the system was brought to 30 μl with ddH_2_O. The PCR amplification program was as follows: 95°C preheating for 3 min; 94°C denaturation for 10 s, 45°C annealing for 20 s, and 72°C extension for 30 s for a total of 30 cycles; and a final extension at 72°C for 5 min. The integrity of DNA was determined using agarose gel electrophoresis, and the concentration of DNA in the sample was quantified with a Qubit3.0 DNA detection kit. Finally, samples were sequenced on the Illumina Miseq platform.

### 2.4 Data processing

The original sequences were processed using the QIIME pipeline (Caporaso et al., 2010). Sequences were classified, and tags, primers, and linker sequences were removed. Next, sequences less than 110 bp in length were removed, as well as ambiguous bases greater than 2 and sequences with an average quality less than 30. Then, chimeras were removed using UCHIME. Finally, UCLUST was used to aggregate the high‐quality sequences into operational taxonomic units, with a similarity threshold of 97%, and to annotate them to the phylum, class, order, family, and genus classification levels, respectively. Rarefaction curves were used to verify whether the number of sequences reflected the microbial diversity of fermented grain samples. As the number of reads increased, the rarefaction curves became more stable, indicating the sequencing data were reasonable and could be used for subsequent analysis.

Heatmap and cluster analyses were performed to identify the dynamic changes in flavor compounds at different fermentation stages and the similarity between samples. The “ComplexHeatmap” package in the R program (Version 4.0.3) was used for data standardization, clustering of samples and variables, and construction of the heatmap.

The Spearman correlation coefficient (*ρ*) was calculated between microorganisms and metabolites, and the connections with *ρ* > .5 and *p* < .05 were selected as visualization objects. For the analysis of coexisting microorganisms, the Spearman correlation coefficient between microorganisms was calculated, and |*ρ*| > .8 and *p* < .05 were selected as interaction objects. Then, data were visualized using Cytoscape 3.4.0 (http://www.cytoscape.org) and Gephi 0.9.3 to characterize the contribution of microorganisms to volatile substances and the impact of physicochemical parameters and microbial interspecies relationships on the core microbiota.

## 3 RESULTS AND DISCUSSION

### 3.1 Multivariate analysis of volatile flavor in fermented grains

**FIGURE 1:**
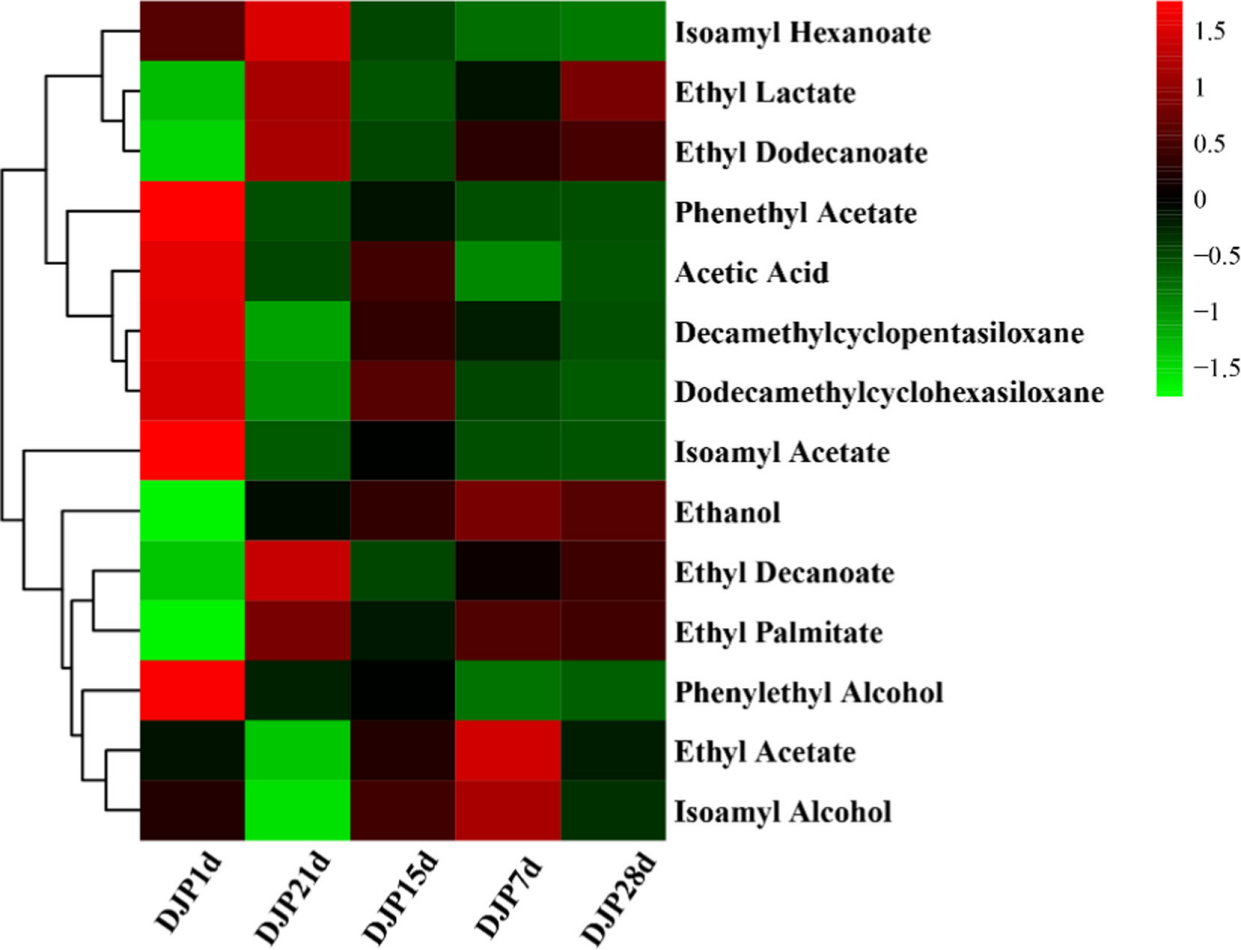
Heatmap based on the change of relative content of metabolites during the fermentation process. DJP1d represents the samples of fermented grains fermented by Daqu for 1 day, corresponding DJP7d for 7 days, DJP15d for 15 days, DJP21d for 21 days, and DJP28d for 28 days.

HS‐SPME‐GC–MS was used to dynamically track and detect the volatile flavor substances in the fermented grains during fermentation, and 14 major volatile products with relative content greater than 1% in at least one sample were identified, including three alcohols, one acid, eight esters, and two alkanes (Figure 1; Table 1). Esters were the most abundant, followed by alcohols, which was consistent with the results of a previous study on the compounds in light‐flavored liquor (Pang et al., 2020). This indicates fermented grains are a direct source of aroma compounds in liquor. Cluster analysis based on the relative content of these compounds revealed three group metabolites: group I contained three esters, namely isoamyl hexanoate, ethyl lactate, and ethyl dodecanoate; group II contained four compounds, namely phenethyl acetate, acetic acid, decamethylcyclopentasiloxane, and dodecamethylcyclohexasiloxane (one ester, one acid, and two alkanes); and group III contained seven compounds (three alcohols and four esters). The esters obtained in this study were all ethyl esters, which is one of the main technical index requirements of distilled liquor. Ethyl esters mainly endow Baijiu with floral and tropical fruit aromas (Lee et al., 2015). Ethanol is the main alcohol in the Baijiu fermentation process, and the relative content of isoamyl alcohol was high, which is associated with brandy aromas and spicy flavors. The content of esters and alcohol metabolites in groups I and III increased after 15 days, indicating a high yield of flavor substances developed during this time period, and the content of esters continued to increase until 28 days. Therefore, appropriately prolonging the fermentation period is an effective strategy to improve the quality of light‐flavored liquor.

**TABLE 1:** Main volatile compounds in fermented grains during fermentation

Number	Keep time (min)	Substances	Relative content (%)				
			DJP1d	DJP7d	DJP15d	DJP21d	DJP28d
1	1.8	Ethanol	7.16	19.62	22.74	21.2	16.92
2	2.55	Ethyl acetate	7.95	9.26	13.58	7.75	3.61
3	3.92	Acetic acid	3.33	2.17	0.76	1.14	1.23
4	4.89	Isoamyl alcohol	12.56	13.11	14.94	11.07	7.87
5	7.7	Ethyl lactate	0.4	0.98	1.38	2.19	2.46
6	11.25	Isoamyl acetate	17.72	5.26	1.23	1.18	0.91
7	18.92	Isoamyl hexanoate	2.31	2.01	1.93	1.91	2.57
8	23.53	Phenylethyl alcohol	10.34	6.21	4.41	4.7	5.7
9	24.24	Decamethylcyclopentasiloxane	2.86	1.76	1.26	0.93	0.43
10	27.53	Phenethyl acetate	5.82	1.21	0.17	0.18	0.19
11	28.69	Dodecamethylcyclohexasiloxane	2.92	1.98	0.87	0.73	0.33
12	30.89	Ethyl decanoate	0.61	3.94	6.11	7.36	11.07
13	35.08	Ethyl dodecanoate	0.34	1.27	1.98	2.15	2.77
14	47.22	Ethyl palmitate	1.64	8.73	11.84	11.44	13.29

*Note*: DJP1d represents the samples of fermented grains fermented by Daqu for 1 day; corresponding DJP7d for 7 days, DJP15d for 15 days, DJP21d for 21 days, and DJP28d for 28 days.

### 3.2 Changes in the physicochemical parameters in fermented grains

**FIGURE 2:**
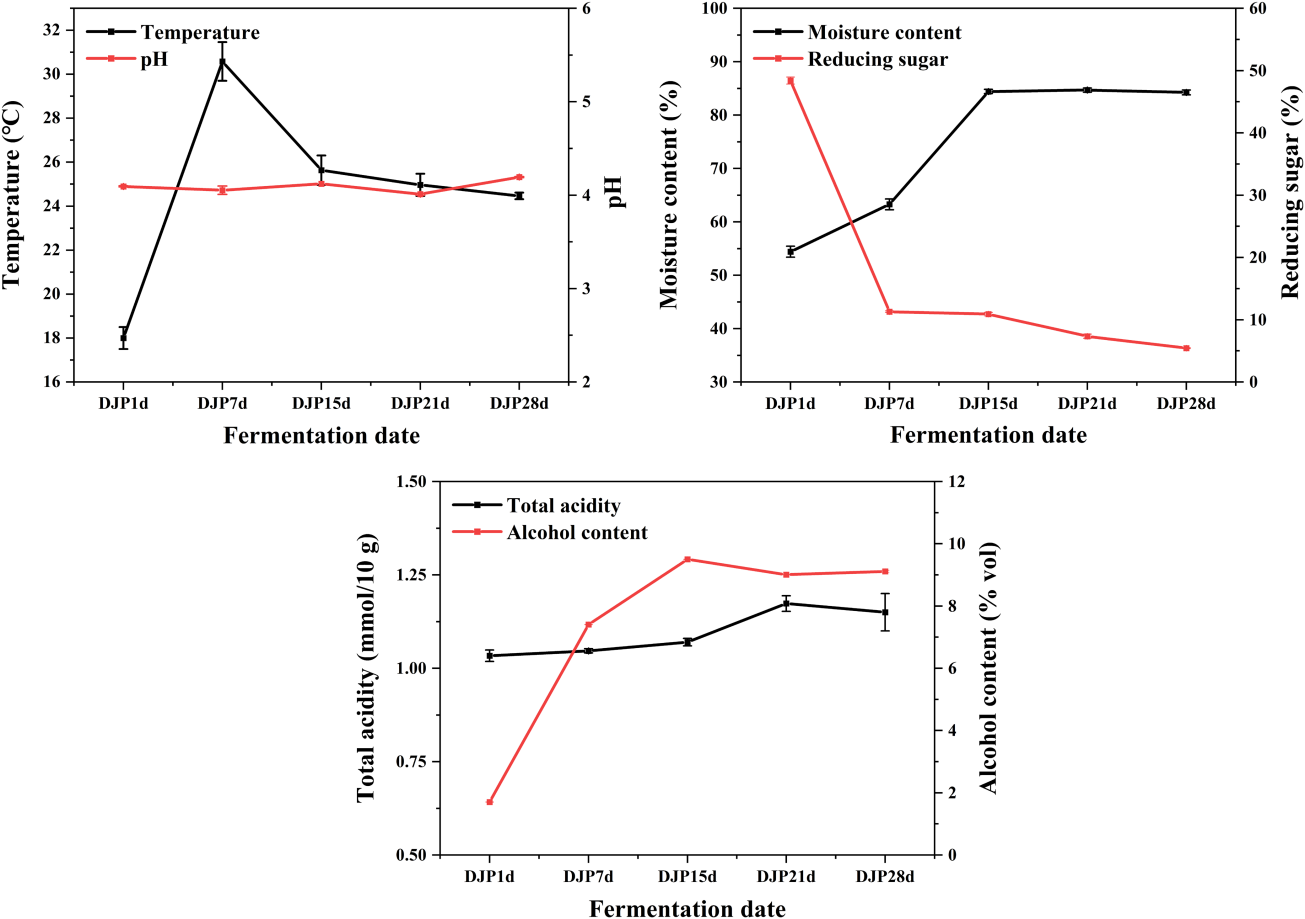
Changes in the major physicochemical factors during the fermentation process. DJP1d represents the samples of fermented grains fermented by Daqu for 1 day, corresponding DJP7d for 7 days, DJP15d for 15 days, DJP21d for 21 days, and DJP28d for 28 days.

The changes in physicochemical parameters of fermented grains are shown in Figure 2. In the early fermentation stage (1–7 days), the moisture content, temperature, and alcohol content increased rapidly, while the reducing sugar significantly decreased. This was due to the massive growth and reproduction of yeast. Most of the reducing sugar was consumed by *Saccharomyces cerevisiae* to generate alcohol. This was accompanied by the production of a large amount of CO_2_ and the consumption of materials, resulting in an increase in moisture content. In addition, microbial metabolism generated heat, leading to a gradual increase in temperature.

In the later stage (7–28 days), the moisture and alcohol content tended to be stable after the initial increase. The reason is, in the later stage of fermentation, the reaction was completed, the material loss was less, and some bacterial cells were decomposed and autolyzed. The change in total acidity was opposite to that of pH throughout the fermentation process. In addition, the reducing sugar and alcohol contents both decreased at first and stabilized later. This is because a small part of reducing sugar was continuously utilized to produce alcohol, while some anaerobic microorganisms use alcohol to grow. In addition, alcohol underwent esterification reactions with acetic acid, resulting in further alcohol consumption.

During the fermentation process of fermented grains, the temperature changes showed a trend of slowly increased in the early stage, rapidly increased in the middle stage, and slowly declined in the later stage. Generally, fermentation requires 6–7 days, and 28–30°C is the most appropriate fermentation temperature. At this time, the fermented grains begin to enter the main fermentation period. After the fermentation temperature reaches the maximum and remains for about 3 days, the postfermentation period, which is the main period of aroma production, is largely over. At this time, the fermentation temperature decreased, and the temperature of the fermented grains was kept at about 24°C when the fermented grains were removed from the tank.

### 3.3 Dynamic analysis of the microbial community in fermented grains

#### 3.3.1 Bacterial community structure

As shown in Table 2, the coverage of each sample reached 100%, indicating all bacterial taxa could be detected, and the sequencing results accurately reflected the samples. The Shannon and Chao indices showed the variation in bacterial diversity of five fermented grain samples was DJP1d > DJP7d > DJP15d > DJP28d > DJP21d. The results indicate the microbial diversity, the number of species, and the richness of the bacterial community decreased gradually as fermentation progressed.

**TABLE 2:** Statistical analysis of bacterial alpha diversity per sample

Sample	Number of reads	OTUs	Shannon	Chao	ACE	Simpson	Coverage (%)
DJP1d	45,165	155	3.10	168.33	164.31	0.09	1.00
DJP7d	38,938	133	3.02	140.09	140.62	0.08	1.00
DJP15d	38,336	120	2.73	123.75	124.93	0.11	1.00
DJP21d	73,695	105	0.65	123.75	126.82	0.80	1.00
DJP28d	45,997	120	1.11	141.23	173.13	0.67	1.00

*Note*: DJP1d represents the samples of fermented grains fermented by Daqu for 1 day, corresponding DJP7d for 7 days, DJP15d for 15 days, DJP21d for 21 days, and DJP28d for 28 days.

Abbreviations: ACE, abundance‐based coverage estimator; OTUs: operational taxonomic units.

**FIGURE 3:**
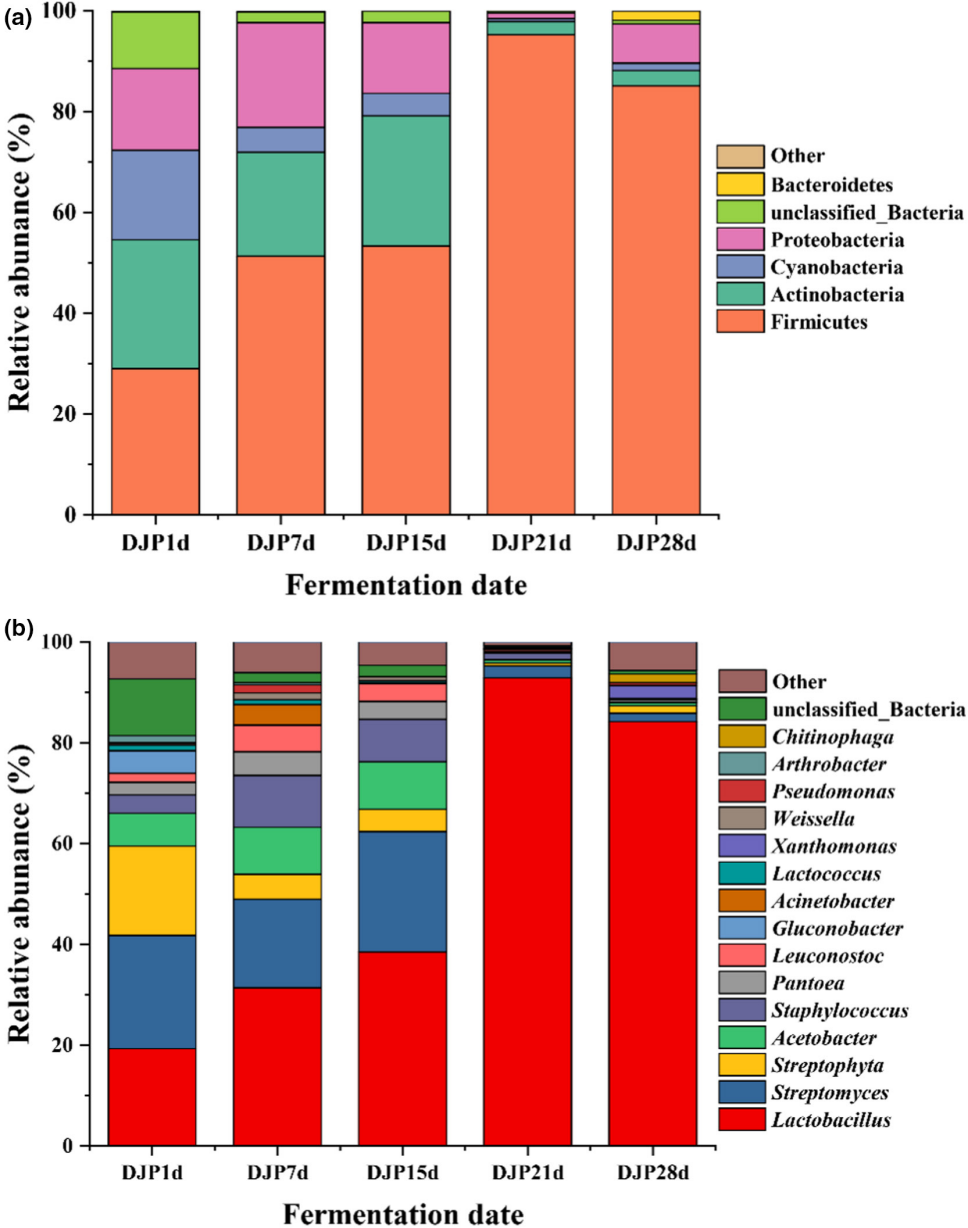
Changes in the relative abundance of bacterial community structure at the phylum (a) and genus (b) levels during the fermentation process. DJP1d represents the samples of fermented grains fermented by Daqu for 1 day, corresponding DJP7d for 7 days, DJP15d for 15 days, DJP21d for 21 days, and DJP28d for 28 days.

For the microbial community composition, among the 12 bacterial phyla detected in the five fermented grain samples, the relative abundance of five phyla exceeded 1% in at least one sample. As shown in Figure 3a, the relative abundance of four bacterial phyla in samples DJP1d, DJP7d, and DJP15d was greater than 1%, including Firmicutes, Actinobacteria, Cyanobacteria, and Proteobacteria, and the sum of their relative abundances accounted for 84.49%, 97.61%, and 97.61% of the three samples, respectively. The relative abundance of three bacterial phyla in sample DJP21d was greater than 1%, including Firmicutes, Actinobacteria, and Cyanobacteria, and the sum of their relative abundance was 99.04%. The relative abundance of five bacterial phyla in sample DJP28d was greater than 1%, including Firmicutes, Actinobacteria, Cyanobacteria, Proteobacteria, and Bacteroidetes, and the sum of their relative abundances was 97.36%.

Among the 128 bacterial genera detected in five fermented grain samples, the relative abundance of 16 genera exceeded 1% in at least one sample. As shown in Figure 3b, the relative abundance of 10 bacterial genera in sample DJP1d was greater than 1%, including *Streptomyces*, *Lactobacillus*, *Streptophyta*, *Acetobacter*, and six other bacterial genera, with relative abundances ranging from 1.08% to 4.46%, and the sum of their relative abundances was 99.44%. The relative abundance of 11 bacterial genera in sample DJP7d was greater than 1%, including *Lactobacillus*, *Streptomyces*, *Staphylococcus*, *Acetobacter*, *Leuconostoc*, *Streptophyta*, and five other bacterial genera, with relative abundances ranging from 1.28% to 4.73%, and the sum of their relative abundances was 92.41%. The relative abundance of eight bacterial genera in sample DJP15d was greater than 1%, including *Lactobacillus*, *Streptomyces*, *Acetobacter*, *Staphylococcus*, *Streptophyta*, *Leuconostoc*, *Pantoea*, and unclassified bacteria, and the sum of their relative abundances was 94.02%. The relative abundance of three bacterial genera in sample DJP21d was greater than 1%, including *Lactobacillus*, *Streptomyces*, and *Staphylococcus*, and the sum of their relative abundances was 96.56%. The relative abundance of five bacterial genera in sample DJP28d was greater than 1%, including *Lactobacillus*, *Xanthomonas*, *Chitinophaga*, *Streptomyces*, and *Streptophyta*, and the sum of their relative abundances was 97.25%.

Firmicutes and *Lactobacillus* were identified as the most abundant bacteria during the fermentation of light‐flavored liquor, which was consistent with the results revealed in previous studies (Pang et al., 2018). However, a significant difference between this work and previous studies was the prokaryotic microbial community structure in fermented grains at different fermentation stages. For example, the maximum relative abundances of Firmicutes (95%) and *Lactobacillus* (92%) at 21 days were much higher than the maximum relative abundances detected in fermented grains at 45 days in a previous study (Firmicutes: 66%, *Lactobacillus*: 27%; Hu et al., 2021).

#### 3.3.2 Fungal community structure

As shown in Table 3, the coverage of each sample was higher than 0.99, indicating most fungal taxa were detected, and the sequencing results accurately reflected the samples. The variations in fungal diversity followed the trend DJP21d > DJP28d > DJP15d > DJP7d > DJP1d, indicating that fungal diversity increased first and then decreased with fermentation time. The diversity reached the highest value at 21 days. However, the changes in the richness of the fungal community were relatively complex and had no obvious regularity.

**TABLE 3:** Statistical analysis of fungal alpha diversity per sample

Sample	Number	OTUs	Shannon	Chao	ACE	Simpson	Coverage
DJP1d	54,999	37	1.23	50.75	65.67	0.32	0.99
DJP7d	60,408	71	1.56	92.38	94.20	0.34	0.99
DJP15d	56,243	71	1.63	80.10	84.27	0.30	0.99
DJP21d	88,758	214	2.03	226.67	222.47	0.22	0.99
DJP28d	57,898	77	1.78	81.50	82.03	0.25	0.99

*Note*: DJP1d represents the samples of fermented grains fermented by Daqu for 1 day, corresponding DJP7d for 7 days, DJP15d for 15 days, DJP21d for 21 days, and DJP28d for 28 days.

Abbreviations: ACE, abundance‐based coverage estimator; OTUs: operational taxonomic units.

**FIGURE 4:**
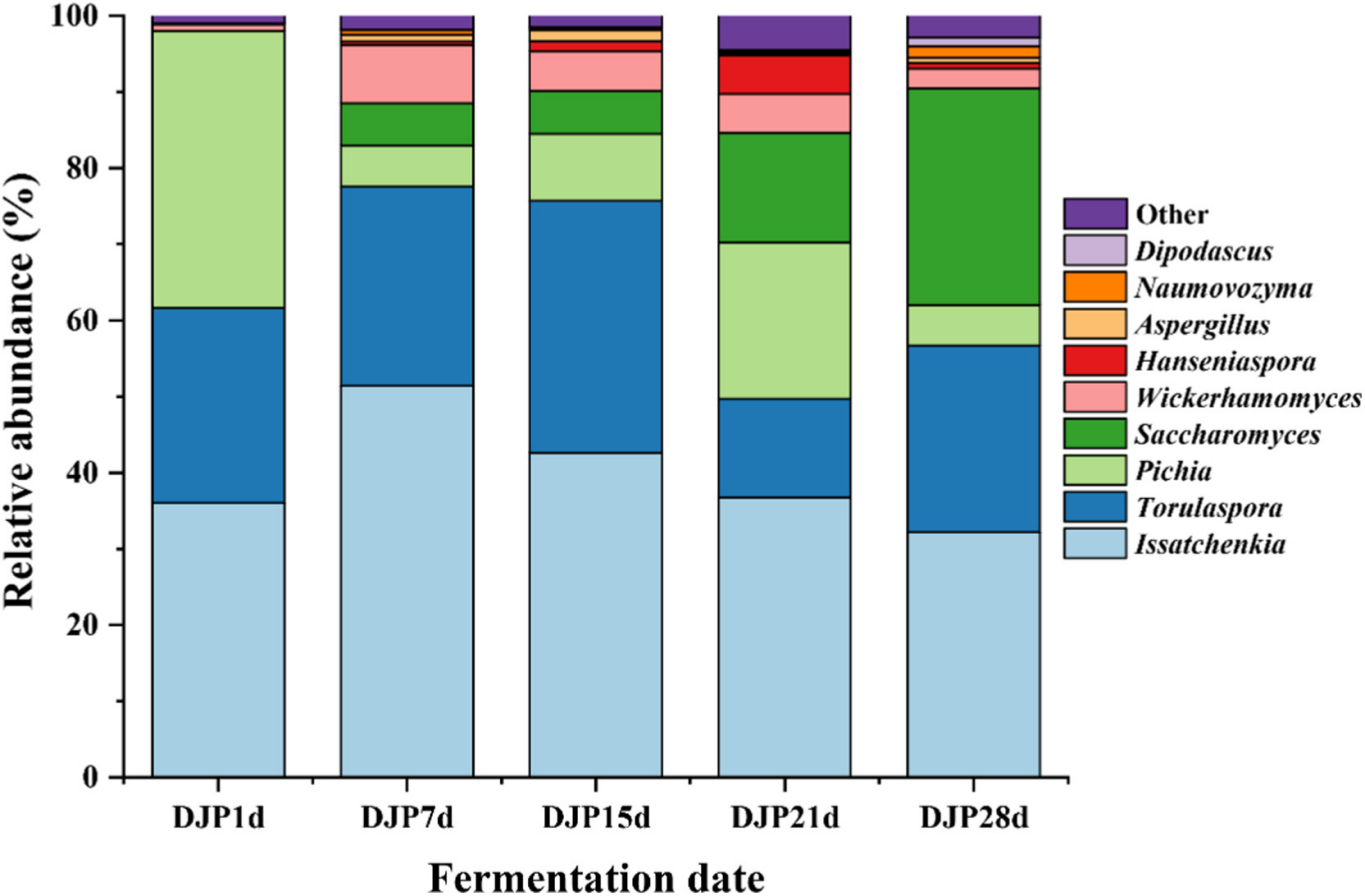
Changes in the relative abundance of fungal community structure at the genus level during the fermentation process. DJP1d represents the samples of fermented grains fermented by Daqu for 1 day, corresponding DJP7d for 7 days, DJP15d for 15 days, DJP21d for 21 days, and DJP28d for 28 days.

As shown in Figure 4, a total of eight fungal phyla were detected in five samples during the fermentation, of which three phyla were common. Their relative abundance accounted for more than 99.49% of each sample. More unique phyla were detected in the DJP21d sample, and the sum of the relative abundance of four phyla only accounted for 0.51%, indicating the phyla that were common between samples were dominant in each sample. In addition, *Ascomycota* was dominant in the five samples (relative abundance accounted for 98.67%–99.97%). Studies have shown *Ascomycota* is the main fungus in the fermented grains of sauce‐flavored, strong‐flavored, and light‐flavored liquors, indicating this phylum is the key fungal microflora in the brewing of Chinese liquor.

Among the 134 fungal genera detected in the five fermented grain samples, nine genera are dominant taxa (relative abundance >1%), and their relative abundances accounted for 95.47%–99.04% of the microbial community of each sample. There were three dominant genera in sample DJP1d, including *Pichia*, *Issatchenkia*, and *Torulaspora*, and the sum of their relative abundances was 99.04%. There were five dominant fungal genera in sample DJP7d, including *Issatchenkia*, *Torulaspora*, *Wickerhamomyces*, *Saccharomyces*, and *Pichia*, with a total relative abundance of 98.19%. There were seven dominant fungal genera in sample DJP15d, including *Issatchenkia*, *Torulaspora*, *Pichia*, *Saccharomyces*, *Wickerhamomyces*, *Aspergillus*, and *Hanseniaspora*, with a total relative abundance of 98.48%. There were six dominant fungal genera in sample DJP21d, including *Issatchenkia*, *Pichia*, *Saccharomyces*, *Torulaspora*, *Hanseniaspora*, and *Wickerhamomyces*, and their total relative abundance reached 95.47%. There were seven dominant fungal genera in sample DJP28d, including *Issatchenkia*, *Saccharomyces*, *Torulaspora*, *Pichia*, *Wickerhamomyces*, *Naumovozym*, and *Dipodascus*, with a total relative abundance of 97.14%.

### 3.4 Correlation between microbiota and volatile flavor

**FIGURE 5:**
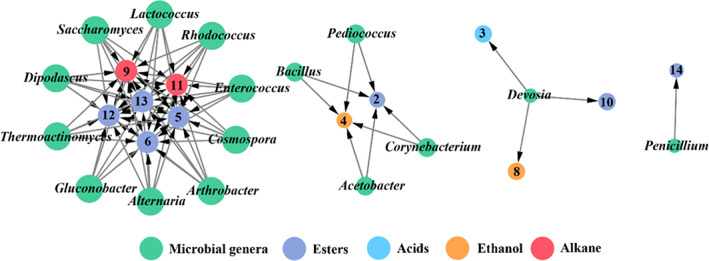
Correlation networks between microbial genera (green) and compounds (other colored circles denote different compound groups) were calculated by Spearman's correlation coefficient (*p* < .5). The size of a node indicates the degree of connectivity. Number 2: Ethyl acetate; number 3: Acetic acid; number 4: Isoamyl alcohol; number 5: Ethyl lactate; number 6: Isoamyl acetate; number 8: Phenylethyl alcohol; number 9: Decamethylcyclopentasiloxane; number 10: Phenethyl acetate; number 11: Dodecamethylcyclohexasiloxane; number 12: Ethyl decanoate; number 13: Ethyl dodecanoate; number 14: Ethyl palmitate

The top 40 bacterial genera and the top 40 fungal genera, in terms of abundance, were selected to calculate the Spearman correlation coefficient between these genera and the 14 major volatile products. The effective connections were identified and selected to draw the network diagram. As shown in Figure 5, a total of 10 genera with high connectivity were identified as the main contributors of metabolites, such as alkanes and esters, and were considered the core microbiota for liquor fermentation. These genera included six bacterial genera, *Lactococcus*, *Rhodococcus*, *Enterococcus*, *Thermoactinomyces*, *Gluconobacter*, and *Arthrobacter*, and four fungal genera, *Saccharomyces*, *Dipodascus*, *Alternaria*, and *Cosmospora*. Figure 5 shows one compound was positively associated with several genera, which is consistent with certain metabolites (such as ethyl lactate and ethyl decanoate) that can only be produced by co‐fermentation of polymicrobial organisms (Kong et al., 2014). This is mainly because the synthetic pathways of certain compounds require enzymes produced by a variety of microorganisms (Hu et al., 2020, 2021). For example, ethyl lactate, ethyl decanoate, and ethyl dodecanoate were positively correlated with fungal genera *Dipodascus*, *Saccharomyces*, *Alternaria*, and *Cosmospora*, and these microorganisms can directly and/or indirectly synthesize the above‐mentioned esters or their precursors. However, three esters were negatively correlated with bacterial genera. In contrast, two alkane substances revealed strong positive correlations with bacterial genera but negative correlations with fungal genera. Moreover, there were some bacterial genera with low connectivity that were significantly positively correlated with acids and ethanol. Therefore, during the fermentation process, the increase in certain fungal biomass can significantly reduce toxic alkanes and increase esters with floral and tropical fruit aromas. However, some bacterial genera, such as *Enterococcus*, are considered to be derived from the animal intestine, plant roots, or soil, and they can contaminate food fermentation (Cai et al., 2013; Gobbetti et al., 2016).

Different fermented foods contain different core microorganisms, such as *Acetobacter* and *Lactobacillus* for the fermentation of vinegar (Li et al., 2016), and *Lactobacillus* and *Streptococcus* for the fermentation of cheese (Monnet et al., 2016). The core microbiota plays an important role in ensuring the flavor diversity of fermented foods and the batch stability of product quality (Awasthi et al., 2014). This study revealed a total of 10 core microbial genera from the fermentation of sorghum grains. For example, *Saccharomyces* is one of the dominant yeast genera in liquor brewing (Wu et al., 2013), capable of producing various flavor esters, ethanol, and other organic acids (Wu et al., 2012). It has been reported that *Lactococcus* exhibits aminopeptidase activity and proteolytic power, and it contributes to the flavor profile of liquor (Centeno et al., 2002). *Thermoactinomyces* can produce abundant enzyme systems, including esterases and phosphatases, which may be involved in the formation of six flavor compounds in liquor, including ethyl lactate, isoamyl acetate, decamethylcyclopentasiloxane, dodecamethylcyclohexasiloxane, ethyl decanoate, and ethyl dodecanoate. *Saccharomyces* has a fermentation activity under anaerobic conditions; it is not only responsible for alcohol production but also for the development of flavor and mouthfeel characteristics of liquor (Tang et al., 2019). In addition, it can tolerate anaerobic and high‐concentration ethanol, interact with other microorganisms such as mold and bacteria, and improve the quality of liquor. Therefore, these taxa were considered the most important vectors that contribute to the development of flavor during fermentation. In addition to the taxa mentioned above, *Bacillus* is also an important bacterium in the production of Baijiu (Wang et al., 2017; Zhang, Wu, et al., 2014). In this study, it promotes the production of ester (ethyl acetate) and alcohol (isoamyl alcohol). Additionally, *Bacillus* can secrete some hydrolases, such as protease, amylase, cellulase, lipase, pectinase, and glucanase, to convert starch into reducing sugar, thereby providing carbon sources for various organisms to improve the esterification rate of fatty acids and alcohols (Hu et al., 2021).

### 3.5 Network of microbial interaction and physicochemical parameters

**FIGURE 6:**
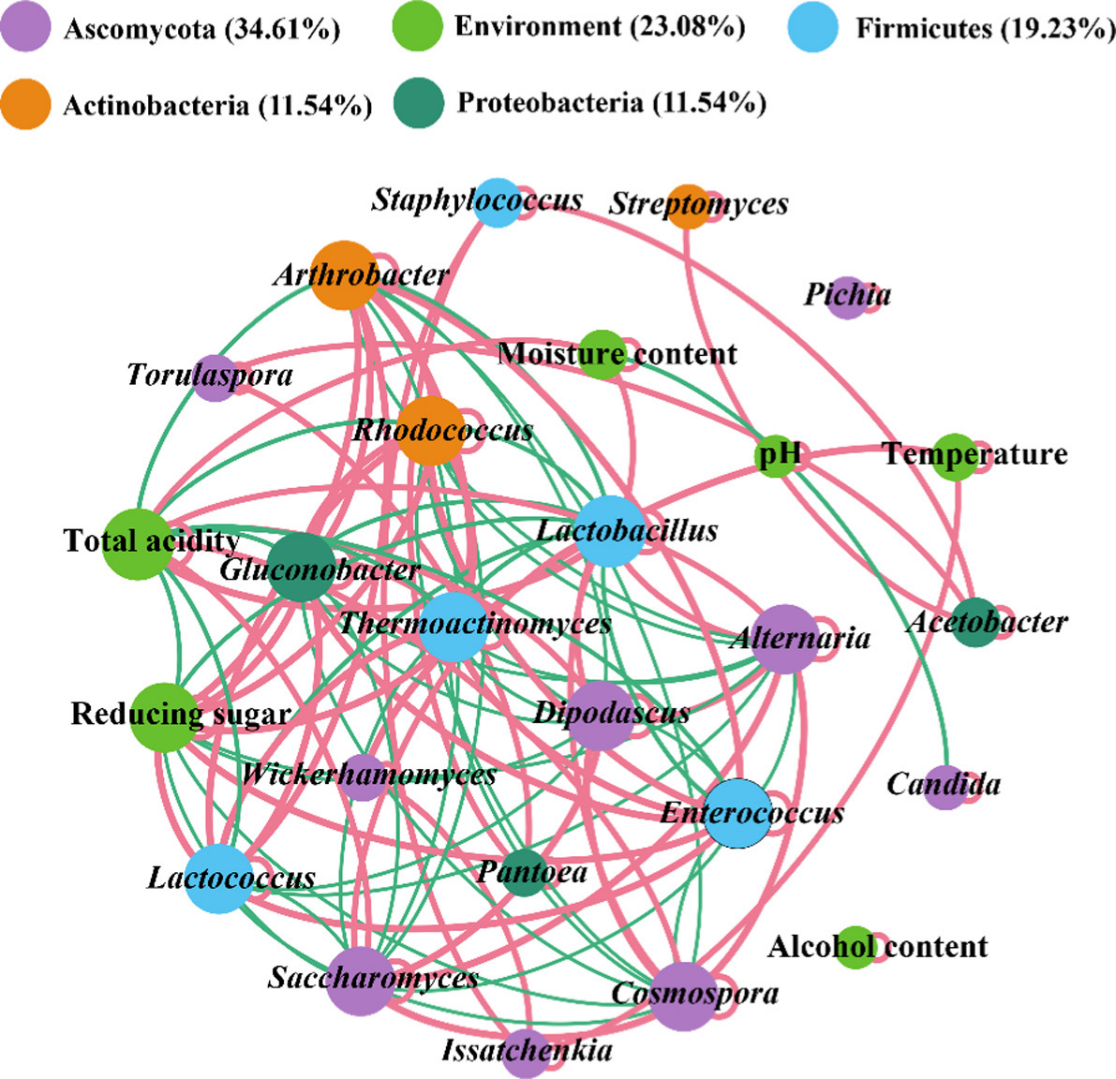
Cooccurrence network of core microbiota, dominant genera, and physicochemical parameters in fermented grains. Nodes of the same color represent the same phyla. The edge thickness is proportional to the value of the Spearman correlation. Red and green edges represent positive and negative interactions between nodes.

By analyzing the network of coexisting microbial genera and metabolites, we obtained 10 taxa with high flavor contributions. These taxa are related to interspecies relationships between microorganisms; however, they are susceptible to environmental factors during fermentation. Therefore, this study selected the core microbiota and dominant bacterial and fungal taxa to explore the impact of microbial interspecies relationships and physicochemical parameters on the core microbiota. As shown in Figure 6, a total of 26 valid connection nodes and 116 edges were obtained. The average clustering coefficient of the network was 0.819, and the modularity index was 5.503. Strongly connected nodes (connections with more than eight edges per node) were mostly distributed in *Ascomycota*, *Actinobacteria*, and *Firmicutes*, suggesting they may help maintain the balance of the fermented grain ecosystem and have potential associations with other microorganisms at the metabolic level during fermentation (Wang, Du, et al., 2018). Two taxa, *Alternaria* and *Arthrobacter*, showed a strong positive correlation with fungal genera and tended to cooperate/mutualism. The taxa were negatively correlated with *Actinobacteria* and *Firmicutes* taxa and tended to be mutually exclusive. This suggests *Alternaria* and *Arthrobacter* have similar roles in regulating metabolite contents during fermentation.

For environmental parameters, total acidity and reducing sugar exhibited high connectivity, and they are important driving factors for the formation of core microbial groups and the succession of dominant groups in fermented grains, thereby indirectly affecting the flavor characteristics. Figure 6 shows total acidity had a significant positive correlation with fungal taxa and a negative correlation with bacterial taxa. In addition, higher reducing sugar promoted the growth of fungi, such as *Arthrobacter* and *Alternaria*, while inhibiting the reproduction of bacteria *Cosmospora* and *Dipodascus*. These results indicate the physicochemical parameters have a strong selective effect on the formation of the microbial community.

Lactic acid bacteria (LAB), facultative anaerobes or anaerobic bacteria, play an important role in regulating the community structure during liquor fermentation. Lactic acid and acetic acid produced by LAB can react with ethanol to form ethyl lactate and ethyl acetate, which are two important flavor substances in liquor (Tao et al., 2014). However, too much ethyl lactate will also generate off‐flavors in light‐flavored liquor. In addition, most LAB could inhibit the growth and reproduction of spoilage microorganisms and pathogenic microorganisms during fermentation due to the antibacterial substances they produce. This suggests bacterial succession may be related to the accumulation of LAB metabolites (Kang et al., 2022). The lactic acid produced from the increase in LAB also reduces the pH value in the fermented grains and affects the community structure. In this study, there were two LAB genera, *Lactobacillus* and *Lactococcus*. *Lactobacillus* is an important microorganism that is essential for the preservation of fermentation food and the enhancement of texture (Zhang et al., 2021). Previous studies have shown *Lactobacillus* may be a biomarker of microbial succession during the fermentation of sorghum grains (Tan et al., 2019). The increase in the abundance of *Firmicutes* during liquor fermentation is mainly related to the increased abundance of *Lactobacillus* (Fan et al., 2019; Zhang et al., 2017). In this study, *Lactobacillus* was highly positively correlated with total acidity and some fungal taxa but negatively correlated with many bacterial taxa, especially *Arthrobacter*, *Enterococcus*, and *Thermoactinomyces*, belonging to Firmicutes. In previous studies on the microbial ecology of liquor fermentation, *Staphylococcus* has been reported (Wang, Zhong, et al., 2018). The main source of *Staphylococcus xylosus* in mature Daqu was the raw materials (Du et al., 2019). Food‐derived *Staphylococcus* spp. strains (i.e., *S. carnosus*, *S. equorum*, *S. succinus*, and *S. xylosus*) are considered safe for use as a starter in food fermentation. They contribute to improving organoleptic properties, including the color, flavor, and taste of fermented foods (Heo et al., 2020). The network analysis revealed that *Staphylococcus* and core taxa tended to cooperate and jointly promote the flavor of liquor. In addition, some dominant and functional fungi were identified, such as *Issatchenkia*, which are abundant in soil and fermented grains and can produce acid‐resistant and high‐temperature‐resistant ethanol and ethyl acetate. These fungi have been reported in many Daqu liquors and different flavor types of fermented grains (Wang, Du, et al., 2018; Wu et al., 2014). *Wickerhamomyces* can produce various esters and aromatic compounds during fermentation (Li et al., 2011; Wang, Du, et al., 2018). The network analysis indicated both fungal genera were strongly influenced by temperature. Previous studies have shown *Candida* is an abnormal microorganism during fermentation of light‐flavored liquor (Li et al., 2011). *C. humilis* and strictly heterologous fermented LAB can convert ethanol production to acetate formation in type I sourdough (De Vuyst et al., 2016). Figure 6 shows *Candida* was negatively correlated with moisture content. This seems to demonstrate that the environmental microbiota can drive microbial succession during fermentation, subtly shaping the quality of liquor.

In summary, the changes in physicochemical parameters, such as total acidity, reducing sugar, and temperature, during the fermentation of liquor affected the reproduction and metabolism of microorganisms, and these environmental conditions were also affected by microbial reproduction and metabolism. Therefore, the combination of microbial interspecies relationships with physical and chemical indicators is of great significance for elucidating the mechanism of flavor development in liquor and improving the quality and fermentation efficiency of light‐flavored liquors.

## 4 CONCLUSION

In conclusion, this study characterized the microbial community composition in sorghum‐based fermented grains at different fermentation periods and quantified the impact of various environmental factors on the core microbial community. The results demonstrated that *Lactobacillus*, *Streptomyces*, *Acetobacter*, *Staphylococcus*, *Pantoea*, and *Leuconostoc* were the main bacterial taxa, and *Issatchenkia*, *Torulaspora*, *Pichia*, *Saccharomyces*, *Wickerhamomyces*, and *Hanseniaspora* were the main fungal taxa. In the early stage of fermentation (1–7 days), the temperature, moisture, and alcohol content showed an upward trend, while the reducing sugar showed a downward trend. As the temperature decreased in the middle and later stages (7–28 days), the physicochemical parameters stabilized. In addition, 14 major volatile products, with relative contents greater than 1% in at least one sample, were identified, including three alcohols, one acid, eight esters, and two alkanes. Network analysis identified a total of 10 core microbiota that could significantly influence the production of ester and alkane volatiles. Moreover, total acidity and reducing sugar played important roles in promoting the formation of the core microbiota and the succession of dominant taxa. These results not only expand our knowledge of the factors affecting liquor quality but also provide new strategies for cultivating new superior strains. We believe the quality of liquor can be stabilized by controlling the physicochemical variations that affect the succession of microbial communities and the associated microorganisms that produce key flavor components.

## CONFLICT OF INTEREST

The authors declare no conflict of interest.

## Data Availability

The data that support the findings of this study are available from the corresponding author [Aiguo Luo] upon reasonable request.
